# Impact of prenatal sirolimus on cardiac rhabdomyomas and brain tubers

**DOI:** 10.1002/uog.70185

**Published:** 2026-02-12

**Authors:** S. Vergote, L. Van der Veeken, D. Chitayat, E. Jaeggi, G. Ryan, E. Miller, S. Shinar

**Affiliations:** ^1^ Fetal Medicine Unit, Department of Obstetrics & Gynaecology Mount Sinai Hospital and University of Toronto Toronto ON Canada; ^2^ Department of Development and Regeneration KU Leuven Leuven Belgium; ^3^ Department of Obstetrics and Gynaecology University Hospitals Antwerp Antwerp Belgium; ^4^ The Prenatal Diagnosis and Medical Genetics Program, Department of Obstetrics and Gynecology, Mount Sinai Hospital University of Toronto Toronto ON Canada; ^5^ Division of Clinical and Metabolic Genetics, Department of Pediatrics, The Hospital for Sick Children University of Toronto Toronto ON Canada; ^6^ Department of Diagnostic Imaging and Interventional Radiology, The Hospital for Sick Children University of Toronto Toronto ON Canada

**Keywords:** mTOR inhibitor, prenatal intervention, rapamycin, rhabdomyoma, SEGA, sirolimus, subcortical tuber, subependymal giant cell astrocytoma, tuberous sclerosis complex

## Abstract

**Objective:**

To document the natural progression of fetal cardiac rhabdomyomas and evaluate the impact of prenatal sirolimus (PNS) on tumor size, cardiac complications and brain‐tuber size.

**Methods:**

This was a single‐center retrospective cohort study of pregnancies with suspected fetal cardiac rhabdomyoma referred to our center from April 2013 to May 2024. Serial ultrasound and echocardiography reports were reviewed to obtain tumor characteristics, such as diameter and location, and cardiac complications, including inflow or outflow obstruction, arrhythmia and hydrops. The tumor‐to‐femur length (TFL) ratio was calculated to correct for fetal growth. Prenatal neurosonography reports were collected from clinical records. Pre‐ and postnatal brain magnetic resonance imaging (MRI) scans were reviewed by two observers who were blinded to PNS treatment. Brain‐lesion severity was assessed based on the presence of subependymal nodules, the EPISTOP score, the maximum diameter of the largest subcortical tuber, the maximum diameter of the largest subependymal giant cell astrocytoma (SEGA) and the diameter of the largest lateral ventricle. Prenatal or postnatal genetic testing results were documented when available.

**Results:**

Twenty‐seven pregnancies were included in the study, seven of which received PNS. Prior to initiation of treatment, the diameter of the largest cardiac tumor at diagnosis was similar between the non‐PNS and PNS groups (mean, 15.2 mm *vs* 14.3 mm; *P* = 0.72), with the left ventricle the most frequently affected location. Without treatment, rhabdomyomas grew rapidly from 20 + 0 to 27 + 6 weeks' gestation (mean, 2.58 mm/week) but growth slowed after 28 weeks (mean, 0.44 mm/week). Hydrops was reported in four cases and occurred at a mean tumor diameter of 31.7 ± 6.2 mm, mean TFL ratio of 0.68 ± 0.15 and at a mean growth rate of 4.3 ± 1.7 mm/week. In the seven women treated with PNS, treatment for > 7 days (*n* = 3) resulted in tumor regression and/or resolution of outflow obstruction, and a reduction in TFL ratio; however, prenatal cessation of treatment resulted in rebound growth (*n* = 2). Treatment for ≤ 7 days (*n* = 4) did not impact tumor size or resolve existing cardiac complications. Among the 12 cases that underwent prenatal MRI, the median EPISTOP score was 7 (interquartile range (IQR), 1–15) and the median largest lateral ventricle diameter was 8.9 (IQR, 7.0–9.7) mm; subcortical tubers and subependymal nodules were each identified in 67% of cases, and SEGAs were identified in 58%. Among the 13 cases that underwent postnatal MRI, the median EPISTOP score was 14 (IQR, 3–16) and the median largest lateral ventricle diameter was 7 (IQR, 7–8) mm; brain tubers were identified in 92% of cases. In cases with both pre‐ and postnatal MRI findings who received PNS for > 7 days (*n* = 3), on postnatal MRI compared with prenatal MRI, one patient showed no change in findings, one demonstrated a mild increase in the largest subcortical tuber diameter and one had no detectable brain tubers.

**Conclusions:**

Early and sustained PNS treatment was associated with reduced cardiac rhabdomyoma size and/or resolution of cardiac complications. Rebound tumor growth was observed after discontinuation of treatment. Brain tubers appeared unchanged with PNS treatment, although the sample size was too small to draw a definitive conclusion. © 2026 The Author(s). *Ultrasound in Obstetrics & Gynecology* published by John Wiley & Sons Ltd on behalf of International Society of Ultrasound in Obstetrics and Gynecology.

## INTRODUCTION

Fetal cardiac tumors are rare, with an incidence of approximately 0.14%^1^. Among these, rhabdomyomas are the most common, accounting for 60–80% of cases^2^. They are typically detected in the second trimester and often appear as multiple tumors in the ventricular myocardium, although single tumors can also occur^3^. Although knowledge of their natural history remains limited, small tumors often regress postnatally without causing physiological dysfunction. In contrast, larger lesions can obstruct outflow tracts, impair ventricular function and cause arrhythmias, potentially leading to hydrops or fetal demise^4^, ^5^.

Rhabdomyomas are associated with tuberous sclerosis complex (TSC) in 80–90% of cases, which is an autosomal dominant disorder characterized by benign tumor growth in multiple organs, including the heart, brain, eyes, skin and kidneys^6^, ^7^. Typical brain lesions include cortical tubers, subependymal nodules and subependymal giant cell astrocytomas (SEGAs). These lesions contribute to a range of neurodevelopmental manifestations, including cognitive impairment and epilepsy, the latter being the most prevalent cause of morbidity in TSC^6^, ^8^. Additionally, poor seizure control is correlated strongly with cognitive impairment, developmental delay and autism spectrum disorder^8^.

In 85–90% of cases, TSC is caused by a pathogenic variant in the *TSC1* or *TSC2* gene, which encode the tumor suppressor proteins hamartin and tuberin, respectively^9^. Variants in these genes can lead to dysregulated signaling pathways, most notably the mammalian target of rapamycin (mTOR) pathway, resulting in uncontrolled tumor formation. Recognizing this, mTOR inhibitors, such as sirolimus and everolimus, have been used postnatally to treat symptomatic rhabdomyomas, renal angiomyolipomas and cutaneous angiofibromas, and to reduce the severity of seizures^10^, ^11^. More recently, case reports have explored prenatal sirolimus (PNS) administration as a potential *in‐utero* treatment, showing tumor shrinkage in some cases^7^, ^12^, ^13^. However, indications for PNS remain undefined, and data on its efficacy, safety and impact on the brain‐tuber burden are limited.

The aims of this study were: (1) to document the natural progression of fetal cardiac rhabdomyomas and brain tubers; and (2) to evaluate the impact of PNS treatment on rhabdomyoma size, cardiac complications, brain‐tuber size and maternal tolerance by comparing treated and untreated (non‐PNS) fetuses.

## METHODS

### Study design

This was a single‐center retrospective cohort study conducted at the Ontario Fetal Centre, a collaboration between Mount Sinai Hospital and The Hospital for Sick Children, in Toronto, ON, Canada. Ethical approval was obtained from the Ethics Committee (REB #24‐0204‐C, #17‐0087‐C and #80667). We reviewed fetal echocardiography reports of pregnant women referred to our center for suspected cardiac rhabdomyoma between April 2013 and May 2024. Fetuses were included if they met at least one of the following criteria: (1) a diagnosis of TSC based on the international TSC diagnostic criteria, requiring either a pathogenic *TSC1* or *TSC2* gene variant or the presence of tumors in multiple organs consistent with the disease (Table S1)^14^; or (2) sonographic features in keeping with cardiac rhabdomyoma, including homogeneous, hyperechogenic, large, growing or multiple cardiac lesion(s). We excluded cases in which pre‐ or postnatal assessment determined that the cardiac tumors were not rhabdomyomas.

### Data collection

Clinical records were reviewed to collect maternal, prenatal and postnatal data. Maternal data included demographics and details of PNS use, including gestational age at initiation, treatment duration and any maternal or fetal side effects. At our center, PNS treatment was consistently initiated with a 15‐mg loading dose of sirolimus, followed by a daily maintenance dose of 5 mg, adjusted as needed to maintain a maternal serum sirolimus level of 10–15 ng/mL. This regimen was based on established guidelines for adult solid‐organ transplantation^15^ and supported by previous prenatal case reports showing a reduction in the size of cardiac tumors^12^, ^16^.

Pre‐ and postnatal data included fetal biometry and neonatal biometry, obtained according to established reference standards^17^, ^18^. Serial ultrasound and echocardiography reports were reviewed to document cardiac tumor characteristics, including number of tumors and location and mean diameter of the largest lesion (calculated as the average of two orthogonal measurements). A change of ≥ 3 mm in tumor diameter was considered significant for classifying the tumor as either growing or shrinking during pregnancy, while a change of < 3 mm was considered stable. To account for fetal growth, we included the tumor‐to‐femur length (TFL) ratio as an exploratory parameter. This ratio was calculated by dividing the mean diameter of the largest tumor by the femur length, which was chosen for its low susceptibility to measurement errors, particularly in the presence of hydrops. Cardiac complications, such as left or right ventricular outflow tract obstruction, atrioventricular or mitral valve inflow obstruction, arrhythmia and hydrops, were documented.

Prenatal and postnatal brain magnetic resonance images were reviewed by two assessors (S.V. and E.M.), who were blinded to PNS exposure and clinical outcome. The reviewers assessed the presence, location and diameter of brain lesions. Prenatal magnetic resonance imaging (MRI) scans were reviewed before postnatal MRI scans to maintain blinding to postnatal findings. Cortical‐tuber burden was quantified using a scoring system previously applied in a study by the EPISTOP consortium, in which fetal MRI lesion sum scores were shown to be associated with neurodevelopmental impairment at 2 years of age^19^. This scoring system (EPISTOP score) assesses cortical and subcortical lesions in the eight cerebral lobes (frontal, temporal, parietal, occipital) and in the two cerebellar hemispheres. Scores are given as 0 (no or doubtful lesions), 1 (single small lesion) or 2 (multiple small lesions or at least one large lesion > 5 mm) in each lobe, with a maximum score of 20. Additionally, for each fetus we recorded the maximum diameter of the largest subcortical tuber, the maximum diameter of any SEGA (defined as a subependymal nodule located near the foramen of Monro; in cases of bilateral SEGA, the largest lesion was measured), the presence of subependymal nodules and the diameter of the largest lateral ventricle.

Prenatal neurosonography reports were collected from clinical records. Postnatal data, including delivery details, gestational age at delivery and neonatal outcomes prior to discharge, were obtained from hospital records, and discharge letters were reviewed to identify early complications or seizures. Lastly, prenatal or postnatal genetic testing results were documented when available.

### Statistical analysis

Statistical analysis was performed using Prism for Windows version 10.4.1 (GraphPad Software, San Diego, CA, USA) and RStudio version 2024.12.1 (Posit PBC, Boston, MA, USA). For continuous variables, normality was assessed using the Shapiro–Wilk test, and data are presented as mean ± SD or median (interquartile range (IQR)), as appropriate. Group comparisons were performed using the unpaired Student's *t*‐test or the Mann–Whitney *U*‐test. Categorical variables are reported as *n* (%) and were analyzed using Pearson's chi‐square test or Fisher's exact test, as appropriate. A two‐tailed *P*‐value of < 0.05 was considered statistically significant.

## RESULTS

Twenty‐seven pregnancies met the inclusion criteria and were included in the study. Of these, 23 cases met the international TSC diagnostic criteria based on a confirmed *TSC1*/*TSC2* pathogenic variant. The remaining four cases were included based on sonographic features, all of which were characteristic of cardiac rhabdomyoma: two cases had multiple rhabdomyomas and two had a single, large, homogeneous, growing mass in the interventricular septum.

Among the 27 women, 20 had normal follow‐up without PNS, while seven patients received PNS treatment. The indication for PNS and the clinical course during treatment are detailed in Table 1. Four cases received PNS for ≤ 7 days, while three received treatment for > 7 days (ranging from 32 to 79 days).

**Table 1:** Indications for prenatal sirolimus (PNS) treatment and pre‐ and postnatal outcomes in seven fetuses with cardiac rhabdomyoma

Case	GA at PNS initiation (weeks)	Duration of PNS treatment (days)	Reason for treatment initiation	Reason for treatment discontinuation	Prenatal outcome	Postnatal outcome	Genetic testing
1	31 + 4	32	Increase in tumor size, reduced LV/RV function	In anticipation of delivery	Tumor regression, LV/RV function improvement	Subclinical seizures	*TSC2* variant
2	24 + 4	6	Increase in tumor size, outflow obstruction	Hydrops	TOP	—	*TSC2* variant
3	30 + 4	2	Outflow obstruction, reduced LV/RV function, arrhythmia, hydrops	Emergency delivery	Emergency delivery for persistent bradycardia	Neonatal death (day 11 after delivery)	Not performed
4	23 + 4	79	Increase in tumor size, outflow obstruction	In anticipation of delivery	Tumor regression, reduced outflow obstruction	No complications	No *TSC1/2* variant
5	26 + 3	2	Increase in tumor size, reduced LV/RV function	Hydrops	TOP	—	*TSC1* variant
6	35 + 2	3	Large tumor, reduced LV function	Not documented	No change	No complications	*TSC2* variant
7	25 + 5	79	Outflow obstruction	Vaginal delivery	No change in tumor size but outflow obstruction resolved	No complications	*TSC2* variant

GA, gestational age; LV, left ventricular; RV, right ventricular; TOP, termination of pregnancy.

### Cardiac outcomes

#### Natural history

**Figure 1:**
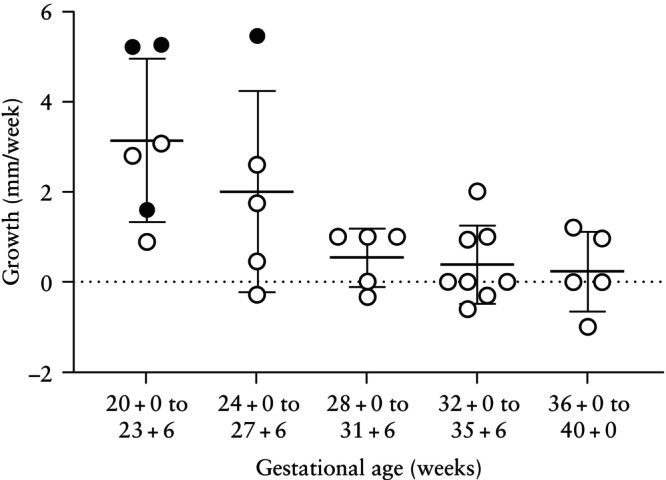
Growth rate of the mean diameter of the largest cardiac rhabdomyoma across gestation in fetuses prior to or without prenatal sirolimus treatment. Each data point represents the growth rate calculated between two consecutive ultrasound examinations, therefore some fetuses contribute more than one data point owing to additional examinations. Black circles represent evolution to hydrops. Data are displayed as mean ± SD.

The following section includes all 27 women. For those treated with PNS, only data obtained prior to treatment initiation were analyzed. The median gestational age at first echocardiographic examination was 29.1 (IQR, 23.0–34.1) weeks. Twenty‐four (89%) cases had multiple rhabdomyomas and three (11%) had a single lesion. The mean diameter of the largest tumor at the initial fetal echocardiographic assessment was 14.6 ± 5.9 mm and the mean TFL ratio was 0.28 ± 0.13. In almost half of the cases (13/27 (48%)), the largest tumor was located in the left ventricle. Other locations were the right ventricle in 26% (*n* = 7), the interventricular septum in 22% (*n* = 6) and the atrioventricular septum in 4% (*n* = 1). Twenty‐one cases had more than one sonographic measurement of the largest tumor recorded during pregnancy. Of those, 62% (*n* = 13) of tumors progressed ≥ 3 mm during pregnancy, 38% (*n* = 8) were stable with no change and none regressed ≥ 3 mm. Rhabdomyomas increased in diameter by a median of 3.0 (IQR, 0–9) mm during pregnancy, with an average growth rate of 1.0 (IQR, 0–2.48) mm/week. When untreated with PNS, rhabdomyomas exhibited growth primarily between 20 + 0 and 27 + 6 weeks (mean, 2.58 ± 1.93 mm/week *vs* 0.44 ± 0.76 mm/week beyond 28 weeks) (Figure 1).

#### Prenatal sirolimus

Maternal and fetal characteristics at the time of the first fetal echocardiographic examination for pregnancies later treated with PNS (*n* = 7) and those that were not treated (*n* = 20) are reported in Table 2. On average, women in the PNS group before initiation of treatment underwent their first fetal echocardiographic examination earlier than non‐PNS patients (median, 21.3 (IQR, 20.6–23.9) weeks *vs* 33.0 (IQR, 28.5–35.0) weeks; *P* = 0.005).

**Table 2:** Maternal and fetal characteristics of 27 cases with suspected cardiac rhabdomyoma at the time of first fetal echocardiographic examination, according to whether they were later treated with prenatal sirolimus (PNS)

Characteristic	No PNS (*n* = 20)	With PNS (before treatment) (*n* = 7)	*P*
Maternal age (years)	30.5 ± 4.9	28.8 ± 6.2	0.49
Nulliparous	6 (30)	4 (57)	0.20
Gestational age (weeks)	33.0 (28.5–35.0)	21.3 (20.6–23.9)	0.005
Number of cardiac tumors			0.001
Single	0 (0)	3 (43)	
Multiple	20 (100)	4 (57)	
Mean diameter of largest tumor (mm)	15.2 ± 5.7	14.3 ± 6.1	0.72
Location of largest tumor			0.65
Left ventricle	8 (40)	5 (71)	
Right ventricle	6 (30)	1 (14)	
Interventricular septum	5 (25)	1 (14)	
Atrioventricular septum	1 (5)	0 (0)	
Cardiac complications*			
Hydrops	1 (5)	1 (14)	0.45
Arrhythmia	1 (5)	1 (14)	0.45
Inflow obstruction	1 (5)	2 (29)	0.16
Outflow obstruction	5 (25)	4 (57)	0.18
Prenatal or postnatal genetic testing			0.73
*TSC1* variant	3/19 (16)	1/6 (17)	
*TSC2* variant	15/19 (79)	4/6 (67)	
No *TSC1*/*2* variant	1/19 (5)	1/6 (17)	

Data are given as mean ± SD, *n* (%), median (interquartile range) or *n/N* (%).

* More than one complication could be recorded in single fetus.

PNS treatment was initiated at a mean gestational age of 28.9 ± 4.2 weeks. At that time, the mean diameter of the largest tumor was 28.0 ± 11.7 mm and the TFL ratio was 0.52 ± 0.23 in these seven patients.

**Figure 2:**
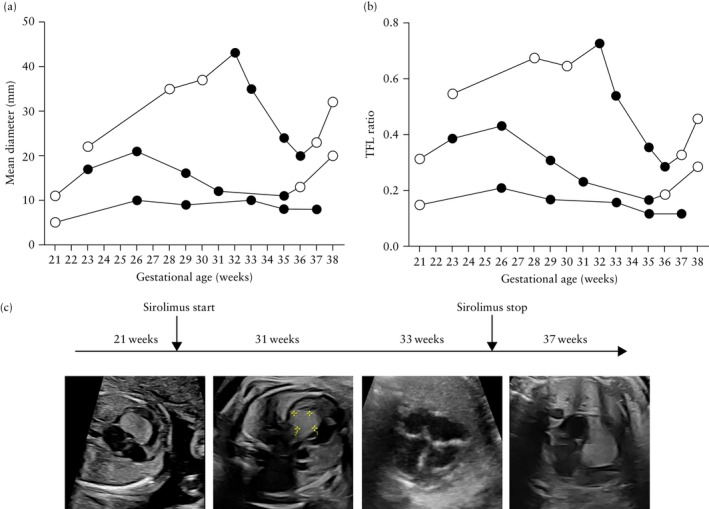
(a,b) Change in size of the largest cardiac rhabdomyoma across gestation in three cases that received prenatal sirolimus (PNS) treatment for > 7 days, assessed by mean tumor diameter (a) and tumor‐to‐femur‐length (TFL) ratio (b). Each line represents an individual case, with black circles representing when patient was treated with PNS. (c) Sequential fetal echocardiographic images from Case 4: at 21 weeks, large rhabdomyoma is visible in the four‐chamber view; by 31 and 33 weeks, there is persistent reduction in tumor size; at 35 weeks, PNS was discontinued; at 37 + 5 weeks, rebound tumor growth was confirmed.

The impact of treatment on tumor size among fetuses who received PNS for > 7 days (*n* = 3) is shown in Figure 2. In two cases, a marked reduction in tumor diameter was observed (from 43 mm to 20 mm between 32 and 36 weeks in one case, and from 21 mm to 11 mm between 26 and 35 weeks in the other), corresponding to a decrease in the TFL ratio. In both of these cases, PNS was discontinued at 36 and 35 weeks, respectively, in anticipation of delivery to minimize the potential risks associated with maternal immunosuppression. However, rebound tumor growth was noted in both cases prior to birth, with tumor diameter increasing from 20 mm to 32 mm and from 11 mm to 20 mm, respectively (Figure 2). In the third case, PNS was initiated owing to outflow obstruction, and treatment continued to delivery. Although tumor diameter remained unchanged, the TFL ratio decreased from 0.21 to 0.12 between 26 and 37 weeks, and the outflow obstruction resolved. Two patients reported a maternal side effect associated with PNS treatment for > 7 days; one patient experienced increased fatigue, and another developed severe oral ulcers at 36 weeks.

Among the four cases that received PNS for ≤ 7 days, one fetus presented at 30 weeks with a large tumor (mean diameter, 28 mm), outflow obstruction, reduced biventricular function, supraventricular tachycardia (SVT) and hydrops. PNS was initiated alongside flecainide (100 mg three times daily) for fetal SVT; however, after 2 days of therapy, an emergency delivery was performed for persistent bradycardia, and the neonate died from multiorgan failure on day 11 after delivery. Two other cases with severe cardiac findings (with the largest tumors measuring 36–38 mm at 25–27 weeks, one of which caused outflow obstruction) developed hydrops after PNS treatment for 2 days and 6 days, respectively. In both cases the parents opted to terminate the pregnancy. In the fourth case that received PNS for ≤ 7 days, the reason for discontinuing PNS was not documented.

#### Cardiac complications

When untreated, hydrops developed in 7% (2/27) of fetuses, arrhythmia in 7% (2/27), inflow obstruction in 11% (3/27) and outflow obstruction in 33% (9/27). After initiation of PNS therapy, two additional fetuses developed hydrops (Table 1).

When considering fetuses with at least one cardiac complication (defined as inflow or outflow obstruction, arrhythmia or hydrops), complications were observed in 11 fetuses at a mean tumor diameter of 19.8 ± 8.6 mm, a mean TFL ratio of 0.42 ± 0.21 and a mean growth rate of 2.9 ± 1.9 mm/week. There was a 20% (1/5) incidence of cardiac complication in fetuses with a mean diameter of the largest tumor ≤ 10 mm, compared to 30% (3/10) for those measuring 11–20 mm and 58% (7/12) for those measuring > 20 mm. No cardiac complications were observed in fetuses with a TFL ratio ≤ 0.2 (0/6), whereas there was a 33% (4/12) incidence of complications in fetuses with a TFL ratio of 0.21–0.4, increasing to 78% (7/9) among those with a TFL ratio > 0.4. Finally, in fetuses with multiple sonographic measurements recorded, those whose tumors did not grow during pregnancy had no cardiac complications (*n* = 0/5). Among those with a growth rate of 0.1–2.0 mm/week, 40% (4/10) developed cardiac complications, compared with 83% (5/6) of those with a growth rate of > 2.0 mm/week.

When focusing only on cases with hydrops (*n* = 4), hydrops occurred at a mean tumor diameter of 31.7 ± 6.2 mm, a mean TFL ratio of 0.68 ± 0.15 and at a mean growth rate of 4.3 ± 1.7 mm/week. All fetuses that developed hydrops had a tumor diameter ≥ 25 mm, a TFL ratio ≥ 0.49 and a growth rate ≥ 1.75 mm/week. In three of the four cases, arrhythmia or inflow/outflow obstruction preceded the onset of hydrops; in the remaining case, hydrops was present at initial presentation alongside outflow obstruction. All hydrops cases resulted in either termination of pregnancy (*n*/*N* = 3/4) or early neonatal death (*n*/*N* = 1/4).

### Brain imaging

Among the 27 pregnancies, 12 underwent prenatal brain MRI at a mean gestational age of 32.5 ± 2.5 weeks. In these cases, the median EPISTOP score was 7 (IQR, 1–15), the median largest lateral ventricle diameter was 8.9 (IQR, 7.0–9.7) mm and brain tubers were identified in 75% (9/12). Among those with brain tubers, subcortical tubers were present in 89% (8/9), subependymal nodules in 89% (8/9) and SEGAs in 78% (7/9). Of the three fetuses without brain tubers observed on prenatal MRI, two had no pathogenic *TSC* variant and had no evidence of brain tubers later in life, while one had a confirmed *TSC2* pathogenic variant and was diagnosed with brain tubers on postnatal imaging.

Of the 15 neonates who survived beyond the perinatal period, 13 underwent postnatal MRI and had images available for review. The median age at postnatal MRI was 3 (IQR, 2–6) days, the median EPISTOP score was 14 (IQR, 3–16) and the median largest lateral ventricle diameter was 7 (IQR, 7–8) mm. Brain tubers were identified in 92% (12/13), including subcortical tuber and subependymal nodule in all cases with brain tubers (100% (12/12)) and SEGA in 92% (11/12).

Table 3 presents the pre‐ and postnatal MRI findings, stratified by PNS exposure for > 7 days *vs* those with no PNS. No significant differences were seen between treatment groups.

**Table 3:** Prenatal and postnatal magnetic resonance imaging (MRI) findings for assessing brain lesion severity, according to prenatal sirolimus (PNS) exposure for > 7 days *vs* untreated

	Prenatal MRI			Postnatal MRI		
Finding on MRI	No PNS (*n* = 9)	PNS (*n* = 3)	*P*	No PNS (*n* = 10)	PNS (*n* = 3)	*P*
Subcortical tuber	6 (67)	2 (67)	1	10 (100)	2 (67)	0.23
Largest diameter (mm)	6.5 (3.4–7.8)	9.5 (7.3–11.8)	0.86	3.9 (2.7–5.9)	11.3 (9.4–13.1)	0.06
EPISTOP score	6 (1–14)	12 (6–14)	0.85	12 (4–16)	16 (8–16)	0.84
Subependymal nodule	6 (67)	2 (67)	1	10 (100)	2 (67)	0.23
SEGA	5 (56)	2 (67)	1	9 (90)	2 (67)	0.42
Largest diameter (mm)	4.0 (3.0–5.5)	7.2 (7.1–7.3)	0.38	5.0 (4.7–6.0)	7.35 (7.2–7.5)	0.29
Bilateral	3/5 (60)	1/2 (50)	1	7/9 (78)	2/2 (100)	1
Largest lateral ventricle diameter (mm)	8.5 (7.0–9.7)	9.5 (8.8–9.7)	0.58	7.0 (6.9–8.0)	9.0 (8.0–9.0)	0.17

Data are given as *n* (%), median (interquartile range) or *n/N* (%). SEGA, subependymal giant cell astrocytoma.

**Figure 3:**
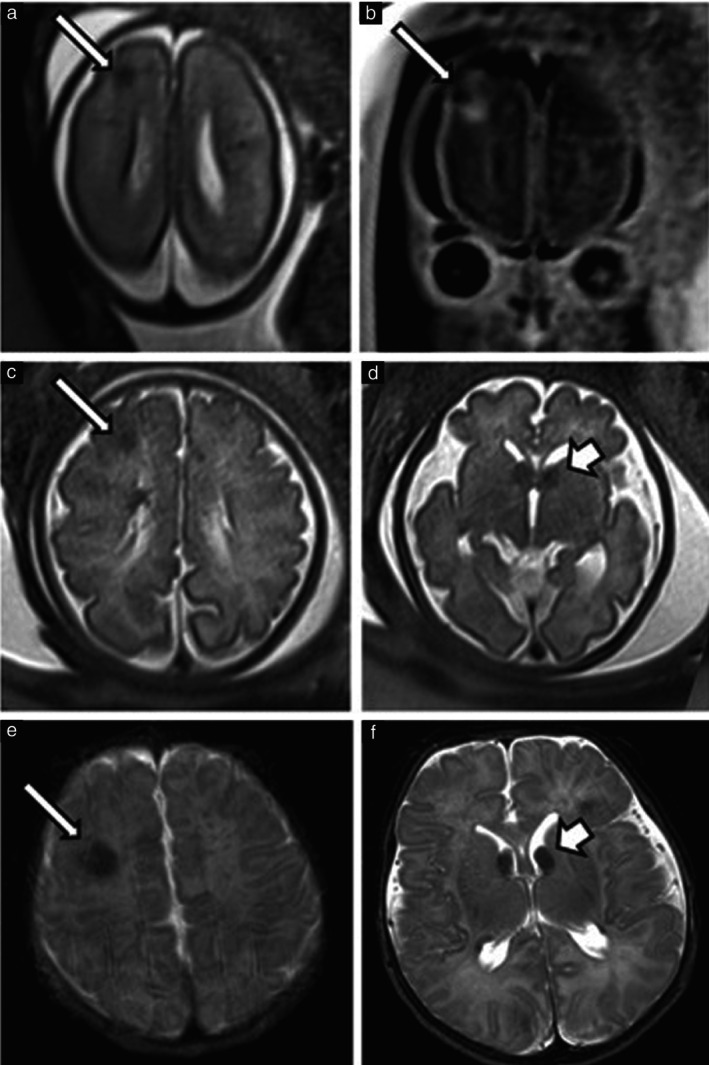
Magnetic resonance imaging (MRI) for assessment of brain lesion severity in single case of tuberous sclerosis complex treated with prenatal sirolimus for > 7 days (initiated at 31 + 4 weeks' gestation). (a,b) Prenatal MRI at 23 weeks showing: (a) axial T2‐weighted image and (b) coronal T2‐weighted image; arrow indicates subcortical tuber. (c,d) Prenatal MRI at 35 weeks showing two axial T2‐weighted images, demonstrating: (c) same subcortical tuber (arrow) as in part (a) and (d) subependymal giant cell astrocytoma (SEGA) (short arrow). (e,f) Postnatal MRI on day 2 after delivery showing two axial T2‐weighted images, demonstrating: (e) the subcortical tuber (arrow) and (f) the SEGA (short arrow), corresponding to the lesions seen prenatally.

Six cases had both prenatal and postnatal MRI, including three cases that received PNS treatment for > 7 days (Table S2). Among the three cases without PNS, all were diagnosed either pre‐ or postnatally with subcortical tubers and SEGAs. In two of these cases, subcortical tuber size and EPISTOP score remained stable; additionally, one had a stable SEGA, while the other showed SEGA growth from 2 mm to 6 mm. The third case had no detectable subcortical tubers on prenatal MRI at 32 weeks' gestation but a 6.2‐mm tuber was observed postnatally (EPISTOP score of 8) and a SEGA was newly diagnosed. In the PNS group, two cases had subcortical tubers, subependymal nodules and SEGAs. One showed no significant change in subcortical tuber size, SEGA size or EPISTOP score. The other had an increase in the largest subcortical tuber diameter (from 5 mm to 7.5 mm), a rise in EPISTOP score (from 12 to 16) and stable SEGA size. The third case had no detectable brain tubers on either pre‐ or postnatal MRI and tested negative for a pathogenic *TSC1*/*2* variant. Figure 3 presents pre‐ and postnatal MRI findings in one case that received PNS treatment for > 7 days.

Of note, six pregnancies underwent both dedicated fetal neurosonography and fetal brain MRI. In four of these cases, brain tubers were identified, while two showed no intracranial lesions. In all six cases, the overall pattern and distribution of lesions observed on neurosonography were concordant with the MRI findings.

### Neonatal outcomes

Of the 27 pregnancies, 11 (41%) resulted in termination of pregnancy, including two that had received PNS for ≤ 7 days, and nine that had not received PNS. Among the remaining 16 pregnancies, all resulted in live birth, although one hydropic fetus exposed to PNS for fewer than 7 days died postnatally following an emergency Cesarean section for abnormal fetal heart rate. Of these 16 deliveries, 11 (69%) were delivered vaginally and five (31%) by Cesarean section.

Among the 11 liveborn infants who were not exposed to PNS, the mean gestational age at delivery was 38.6 ± 1.5 weeks, with no preterm births (< 37 weeks). The median birth weight was 3300 (IQR, 2620–3390) g. Postnatally, 3/11 (27%) infants had subclinical seizures on electroencephalography requiring antiepileptic treatment prior to discharge. Additional findings included two cases of arrhythmia (one with self‐resolving sinus bradycardia and one with new‐onset SVT requiring propranolol), one retinal astrocytoma, one retinal hemorrhage and one case of pulmonary hypertension. The average length of neonatal hospitalization was 6.7 ± 3.1 days.

Three liveborn infants had been exposed to PNS for more than 7 days, of whom two were delivered at 37 weeks and one was delivered at 38 weeks. One of the infants delivered at 37 weeks had a birth weight of 2370 g (7^th^ percentile). This infant was estimated at the 47^th^ percentile at 25 weeks, prior to PNS initiation, but dropped to the 7^th^ percentile by 35 weeks, with otherwise normal Doppler findings. The mother was a primigravida with a body mass index (BMI) of 15.2 kg/m^2^. The other two infants weighed 4340 g and 2790 g at birth (99^th^ and 24^th^ percentiles, respectively). Among these three cases, one neonate experienced multiple subclinical seizures requiring antiepileptic therapy. The other two neonates had an uncomplicated neonatal course. These infants were discharged after 4, 5 and 16 days, respectively.

Two liveborn infants had received PNS for ≤ 7 days. As mentioned, one of these cases had hydrops prior to initiation of PNS treatment at 30 + 4 weeks and was delivered via emergency Cesarean section at 30 + 6 weeks owing to abnormal fetal heart tracing; the neonate died at 11 days of age owing to multiorgan failure. In the other case, PNS was discontinued early for unclear reasons. The infant was delivered vaginally at 40 weeks, had an uncomplicated neonatal course and was discharged 1 day after delivery. All neonates treated with PNS had a normal complete blood cell count after birth, regardless of duration of PNS treatment and continuation to birth.

## DISCUSSION

### Main findings

In this retrospective study of 27 pregnancies, we characterized the natural history of fetal cardiac rhabdomyomas. The median gestational age at first echocardiographic examination was 29.1 weeks, with a mean largest tumor diameter of 14.6 ± 5.9 mm and a mean TFL ratio of 0.28 ± 0.13. Most fetuses had multiple tumors (89%) and a confirmed *TSC1*/*2* variant (23/25 (92%)). Untreated tumors typically grew during pregnancy, particularly between 20 + 0 and 27 + 6 weeks. No cardiac complications occurred in fetuses with a TFL ratio of ≤ 0.2 or non‐growing tumors. PNS decreased tumor size, reduced outflow obstruction and improved biventricular function when initiated early and maintained until delivery; however, rebound tumor growth occurred if treatment was discontinued prematurely in anticipation of delivery. In contrast, PNS appeared ineffective in some cases complicated by hydrops or by large, rapidly growing tumors that had already compromised outflow and cardiac function. PNS did not appear to significantly affect brain‐tuber size, although our small sample size limited the ability to draw definitive conclusions.

### Comparison with literature

Our findings are consistent with observations published previously on the natural history of fetal cardiac rhabdomyomas^7^. In our cohort, the median gestational age at diagnosis was 29.1 weeks, comparable to the 30.5 weeks reported in the meta‐analysis of Mustafa *et al*.^7^, which included 61 studies and 400 fetuses. We observed a smaller mean largest tumor diameter (14.6 ± 5.9 mm) compared with 21 mm in the meta‐analysis, probably reflecting our use of the average of two orthogonal measurements rather than a single maximal diameter. A confirmed diagnosis of TSC was more frequent in our cohort (92% *vs* 60%), possibly reflecting a more consistent application of diagnostic criteria and broader access to molecular testing in our center. Mustafa *et al*.^7^ reported prenatal arrhythmia in 13% of cases, outflow obstruction in 16% and hydrops in 10%, in keeping with our untreated cohort. Prenatal tumor regression was seen in 13% of cases in the meta‐analysis, compared with none in our untreated cohort. This may reflect methodological differences, as the meta‐analysis lacked a defined threshold for tumor reduction, probably because of inconsistent reporting, while we applied a stricter criterion of ≥ 3 mm decrease. Of note, tumor growth appeared to be faster before 28 weeks in our cohort; however, this may reflect selection bias, as rapidly growing tumors were more likely to result in early termination, leaving predominantly slower‐growing cases after 28 weeks.

Regarding PNS, the meta‐analysis of Mustafa *et al*.^7^ included nine fetuses, all showing tumor reduction and resolution of outflow obstruction, with no fetal or neonatal deaths or need for surgery. Our three PNS cases treated for more than 7 days showed similar outcomes, with reductions in tumor size or TFL ratio and improved cardiac function. Interestingly, two cases with significant cardiac‐tumor size reduction under treatment demonstrated rebound growth prenatally after discontinuation of PNS prior to delivery. This phenomenon has also been described in cases of postnatal mTOR use^20^, in which the rebound growth necessitated restarting mTOR inhibition^10^.

Brain‐tuber burden was substantial in our cohort, with identification in 75% of cases on prenatal MRI and in about 90% on postnatal MRI, consistent with the results of prior studies^21^, ^22^. The higher postnatal detection rate probably reflects improved MRI resolution, ongoing brain myelination with reduced water content, and potential lesion growth. We observed no significant difference in brain‐tuber burden between PNS‐treated and untreated fetuses, although the sample size was small. In postnatal TSC studies, everolimus, an mTOR inhibitor similar to sirolimus, has been shown to reduce SEGA volume, with 42% of cases achieving a 50% or greater reduction by 24 weeks of treatment^13^. We did not observe similar effects in our cohort: the duration of therapy may have been too short, fetal and postnatal lesion biology may have differed, the sample size might have been too small to detect meaningful changes or the follow‐up period may have been insufficient to capture the full impact of treatment. Recent evidence indicates that dedicated neurosonography can reliably detect brain tubers in TSC^23^, consistent with our findings of concordant patterns in six cases evaluated using both modalities. We believe that, while neurosonography can detect brain involvement in TSC, MRI provides superior soft‐tissue contrast, allowing visualization of smaller or non‐structural abnormalities such as signal alterations and white matter hyperintensities that are beyond the resolution of ultrasound. Therefore, MRI remains essential for the accurate assessment of lesion burden and distribution.

### Clinical implications

Seven cases in our cohort received PNS, with three cases receiving treatment for > 7 days. In one case without a confirmed *TSC1*/*2* variant, tumor regression was observed, suggesting a potential benefit of sirolimus in TSC variant‐negative fetuses, although alternative explanations such as mosaicism or other etiologies cannot be excluded. In two cases, substantial tumor reduction during treatment was followed by rebound growth when PNS was discontinued before delivery. Although there were only mild maternal side effects in two patients during treatment and no complications in the third patient who remained on PNS therapy until birth, we did observe one infant born at the 7^th^ percentile despite normal Doppler findings. The mother was a primigravida with a BMI of 15.2 kg/m^2^, suggesting constitutional growth restriction, although fetal growth restriction has been reported in another case of fetal rhabdomyoma treated with PNS^24^. There were also two cases with severe cardiac findings (large tumors causing outflow obstruction or impaired left and right ventricular function) that received PNS for 2 and 6 days; both developed hydrops and the parents elected to terminate the pregnancies. Given the pre‐existing cardiac complications and the very short duration of treatment, as well as the possible effect of hydrops on reduced medication penetration, these outcomes are unlikely to reflect a contributory effect of PNS, although this possibility cannot be completely excluded. Based on these findings, we recommend individualized clinical decisions guided by maternal and fetal side effects, but would consider continuing PNS until delivery to reduce the risk of rebound tumor growth.

To better define the optimal treatment window, we examined the timing and characteristics of cardiac complications, as prior studies have linked tumor diameter > 20 mm, arrhythmia and hydrops with adverse neonatal outcomes^5^. In our cohort, cardiac complications occurred in 41% of cases and were typically associated with tumors measuring a mean of 19.8 ± 8.6 mm in diameter, with a mean TFL ratio of 0.42 ± 0.21 and a mean growth rate of 2.9 ± 1.9 mm/week. PNS was usually initiated at the onset of these complications; however, this timing often proved too late, as some fetuses deteriorated rapidly. In four cases in which PNS was discontinued within 7 days, three cases had large, rapidly growing tumors and progressed to hydrops, leading to termination of pregnancy or neonatal death. This suggests that the effectiveness of PNS is limited once hydrops has developed, possibly owing to limited absorption. Although larger tumors carried greater risk, cardiac complications also occurred in 20% of cases with a largest tumor diameter ≤ 10 mm. In contrast, no cardiac complications were seen in fetuses with a TFL ratio of ≤ 0.2 or non‐growing tumors. These findings highlight the potential value of assessing tumor burden relative to fetal size and growth rate. We suggest that initiating PNS before tumor diameter exceeds 20 mm, the TFL ratio exceeds 0.2 or growth surpasses 2 mm/week may be beneficial, acknowledging that these thresholds remain exploratory and are not yet validated as definitive predictors.

### Strengths and limitations

This study has several strengths. We report the largest single‐center series of fetuses treated with PNS following a standardized dosing regimen. Tumor measurements were obtained from expert fetal echocardiography reports and normalized using the TFL ratio. Brain magnetic resonance images were reviewed by a blinded pediatric neuroradiologist and maternal–fetal medicine specialist to reduce measurement bias. To our knowledge, this is the first study to assess the impact of PNS on brain tubers.

Limitations include the small sample size, particularly within the PNS subgroup and among those with both prenatal and postnatal brain imaging, limiting generalizability. Treatment was not randomized, introducing potential indication bias. Long‐term outcomes were unavailable, preventing evaluation of sustained effects on cardiac function, neurodevelopment and seizure control. Finally, this study was not designed to assess the predictive accuracy of size and growth of cardiac rhabdomyomas for prediction of cardiac complications or hydrops, which requires prospective validation.

### Conclusions

We described the natural history of fetal cardiac rhabdomyomas and the timing of associated cardiac complications. Early, sustained PNS treatment reduced tumor size and complications; however, prenatal rebound tumor growth occurred after discontinuation. PNS appeared ineffective in some cases with hydrops or those with large, rapidly growing tumors already compromising cardiac function. In a small subset of patients with prenatally documented brain lesions, PNS had limited impact on brain tubers.

## Supporting information


**Table S1** Diagnostic criteria for tuberous sclerosis complex (TSC)^14^.
**Table S2** Magnetic resonance imaging (MRI) findings in six cases that underwent both prenatal and postnatal imaging assessments.

## Data Availability

The data that support the findings of this study are available from the corresponding author upon reasonable request.

## References

[1] Uzun O , Wilson DG , Vujanic GM , Parsons JM , De Giovanni JV . Cardiac tumours in children. Orphanet J Rare Dis. 2007;2:11. 17331235 10.1186/1750-1172-2-11 PMC3225855

[2] Allan LD , Hornberger L , Sharland GK . Textbook of fetal cardiology. Greenwich Medical Media; 2000.

[3] Yinon Y , Chitayat D , Blaser S , et al. Fetal cardiac tumors: a single‐center experience of 40 cases. Prenat Diagn. 2010;30(10):941‐949. 20721876 10.1002/pd.2590

[4] Okmen F , Ekici H , Hortu I , et al. Outcomes of antenatally diagnosed fetal cardiac tumors: a 10‐year experience at a single tertiary referral center. J Matern Fetal Neonat Med. 2022;35(18):3489‐3494. 10.1080/14767058.2020.1822316 32954877

[5] Chao A , Chao A , Wang T , et al. Outcome of antenatally diagnosed cardiac rhabdomyoma: case series and a meta‐analysis. Ultrasound Obstet Gynecol. 2008;31(3):289‐295. 18307215 10.1002/uog.5264

[6] Curatolo P , Bombardieri R , Jozwiak S . Tuberous sclerosis. Lancet. 2008;372(9639):657‐668. 18722871 10.1016/S0140-6736(08)61279-9

[7] Mustafa HJ , Javinani A , Morning ML , et al. Characteristics and outcomes of fetal cardiac rhabdomyoma with or without mTOR inhibitors, a systematic review and meta‐analysis. Prenat Diagn. 2024;44(10):1251‐1267. 39164800 10.1002/pd.6640

[8] Franz D , Bissler J , McCormack F . Tuberous sclerosis complex: neurological, renal and pulmonary manifestations. Neuropediatrics. 2010;41(5):199‐208. 21210335 10.1055/s-0030-1269906 PMC4629839

[9] Sancak O , Nellist M , Goedbloed M , et al. Mutational analysis of the TSC1 and TSC2 genes in a diagnostic setting: genotype–phenotype correlations and comparison of diagnostic DNA techniques in Tuberous Sclerosis Complex. Eur J Hum Genet. 2005;13(6):731‐741. 15798777 10.1038/sj.ejhg.5201402

[10] Sugalska M , Tomik A , Jóźwiak S , Werner B . Treatment of cardiac rhabdomyomas with mTOR inhibitors in children with tuberous sclerosis complex—a systematic review. Int J Environ Res Public Health. 2021;18(9):4907. 34062963 10.3390/ijerph18094907 PMC8124908

[11] French JA , Lawson JA , Yapici Z , et al. Adjunctive everolimus therapy for treatment‐resistant focal‐onset seizures associated with tuberous sclerosis (EXIST‐3): a phase 3, randomised, double‐blind, placebo‐controlled study. Lancet. 2016;388(10056):2153‐2163. 27613521 10.1016/S0140-6736(16)31419-2

[12] Vachon‐Marceau C , Guerra V , Jaeggi E , et al. In‐utero treatment of large symptomatic rhabdomyoma with sirolimus. Ultrasound Obstet Gynecol. 2019;53(3):420‐421. 30549350 10.1002/uog.20196

[13] Franz DN , Belousova E , Sparagana S , et al. Long‐term use of everolimus in patients with tuberous sclerosis complex: final results from the EXIST‐1 study. PLoS One. 2016;11(6):e0158476. 27351628 10.1371/journal.pone.0158476 PMC4924870

[14] Northrup H , Aronow ME , Bebin EM , et al. Updated international tuberous sclerosis complex diagnostic criteria and surveillance and management recommendations. Pediatr Neurol. 2021;123:50‐66. 34399110 10.1016/j.pediatrneurol.2021.07.011

[15] Costanzo MR , Dipchand A , Starling R , et al. The International Society of Heart and Lung Transplantation Guidelines for the care of heart transplant recipients. Elsevier; 2010:914‐956. 10.1016/j.healun.2010.05.034 20643330

[16] Barnes BT , Procaccini D , Crino J , et al. Maternal sirolimus therapy for fetal cardiac rhabdomyomas. N Engl J Med. 2018;378(19):1844‐1845. 29742370 10.1056/NEJMc1800352 PMC6201692

[17] Hadlock FP , Harrist RB , Sharman RS , Deter RL , Park SK . Estimation of fetal weight with the use of head, body, and femur measurements – a prospective study. Am J Obstet Gynecol. 1985;151(3):333‐337. 3881966 10.1016/0002-9378(85)90298-4

[18] Villar J , Ismail LC , Victora CG , et al. International standards for newborn weight, length, and head circumference by gestational age and sex: the Newborn Cross‐Sectional Study of the INTERGROWTH‐21st Project. Lancet. 2014;384(9946):857‐868. 25209487 10.1016/S0140-6736(14)60932-6

[19] Hulshof HM , Slot EMH , Lequin M , et al. Fetal brain magnetic resonance imaging findings predict neurodevelopment in children with tuberous sclerosis complex. J Pediatr. 2021;233:156‐162.e2. 33640330 10.1016/j.jpeds.2021.02.060

[20] Hurtado‐Sierra D , Ramos Garzón JX , Romero‐Guevara SL , Serrano‐García AY , Rojas LZ . Everolimus and sirolimus in the treatment of cardiac rhabdomyomas in neonates. Pediatr Res. 2025. 10.1038/s41390-025-04043-8. PMC12811118 40287604

[21] Mammadova D , Vecko J , Hofmann M , et al. A single‐center observational study on long‐term neurodevelopmental outcomes in children with tuberous sclerosis complex. Orphanet J Rare Dis. 2023;18(1):349. 37946245 10.1186/s13023-023-02959-0 PMC10637019

[22] Henske EP , Jóźwiak S , Kingswood JC , Sampson JR , Thiele EA . Tuberous sclerosis complex. Nat Rev Dis Primers. 2016;2(1):1‐18. 10.1038/nrdp.2016.35 27226234

[23] Malinger G , Prabhu A , Maroto González A , et al. Fetal neurosonography as accurate tool for diagnosis of brain involvement in tuberous sclerosis. Ultrasound Obstet Gynecol. 2023;62(3):391‐397. 37021742 10.1002/uog.26213

[24] Pluym ID , Sklansky M , Wu JY , et al. Fetal cardiac rhabdomyomas treated with maternal sirolimus. Prenat Diagn. 2020;40(3):358‐364. 31742705 10.1002/pd.5613

