# Supplementary material for: Impact of prenatal sirolimus on cardiac rhabdomyomas and brain tubers
Source: Ultrasound Obstet Gynecol. 2026 Feb 12;67(3):350–9. doi: 10.1002/uog.70185 (PMC12951259; doi:10.1002/uog.70185)
Supplement: Supplementary file 1 — Table S1 Diagnostic criteria for tuberous sclerosis complex (TSC) 14 . Table S2 Magnetic resonance imaging (MRI) findings in six cases that underwent both prenatal and postnatal imaging assessments. [file UOG-67-350-s001.docx]

## Table S1: Diagnostic criteria for tuberous sclerosis complex (TSC)^14^.

| **Major Criteria** | **Minor Criteria** |
| --- | --- |
| Hypomelanotic macules (≥3; at least 5 mm diameter) | “Confetti” skin lesions |
| Angiofibroma (≥3) or fibrous cephalic plaque | Dental enamel pits (≥3) |
| Ungual fibromas (≥2) | Intraoral fibromas (≥2) |
| Shagreen patch | Retinal achromic patch |
| Multiple retinal hamartomas | Multiple renal cysts |
| Multiple cortical tubers and/or radial migration lines | Nonrenal hamartomas |
| Subependymal nodule (≥2) | Sclerotic bone lesions |
| Subependymal giant cell astrocytoma |  |
| Cardiac rhabdomyoma |  |
| LAM (Lymphangioleiomyomatosis) |  |
| Angiomyolipomas (≥2) |  |

- ***Definite TSC:*** *2 major features or 1 major feature with 2 minor features*
- ***Possible TSC:*** *Either 1 major feature or ≥2 minor features*
- ***Genetic diagnosis:*** *A pathogenic variant in TSC1 or TSC2 is diagnostic for TSC. Most TSC-causing variants are sequence variants that clearly prevent TSC1 or TSC2 protein production. Some variants compatible with protein production (e.g., some missense changes) are well established as disease-causing; other variant types should be considered with caution.*

Table S2: Magnetic resonance imaging (MRI) findings in six cases that underwent both prenatal and postnatal imaging assessments.

| **ID** | **Prenatal MRI** | | | | **Postnatal MRI** | | | |
| --- | --- | --- | --- | --- | --- | --- | --- | --- |
|  | **GA at MRI (weeks)** | **Largest subcortical tuber diameter** | **Largest SEGA diameter** | **EPISTOP Score** | **Age at MRI (days)** | **Largest subcortical tuber diameter** | **Largest SEGA diameter** | **EPISTOP Score** |
|  | **No PNS** | | | | | | | |
| **1** | 36+6 | 2.5 | 4 | 14 | 6 | 2.4 | 4.7 | 16 |
| **2** | 32+4 | 7 | 2 | 16 | 6 | 6.7 | 6 | 16 |
| **3** | 31+4 | 0 | 0 | 0 | 2 | 6.2 | 3.4 | 8 |
|  | **PNS >7 days** | | | | | | | |
| **4** | 31+4 | 14 | 7.4 | 16 | 2 | 15 | 7 | 16 |
| **5** | 28+0 | 0 | 0 | 0 | 25 | 0 | 0 | 0 |
| **6** | 29+2 | 5 | 7 | 12 | 7 | 7.5 | 7.7 | 16 |

**Values in mm. EPISTOP score has a maximum score of 20.**
